# Sucrose-Enriched and Carbohydrate-Free High-Fat Diets Distinctly Affect Substrate Metabolism in Oxidative and Glycolytic Muscles of Rats

**DOI:** 10.3390/nu16020286

**Published:** 2024-01-18

**Authors:** Daniel Da Eira, Shailee Jani, Mateja Stefanovic, Rolando B. Ceddia

**Affiliations:** Muscle Health Research Centre, School of Kinesiology and Health Science, York University, Toronto, ON M3J 1P3, Canada

**Keywords:** PGC-1α, TFAM, ketolysis, insulin resistance, anaplerosis, ketogenic diet, OXCT, ACAT1

## Abstract

Skeletal muscle substrate preference for fuel is largely influenced by dietary macronutrient availability. The abundance of dietary carbohydrates promotes the utilization of glucose as a substrate for energy production, whereas an abundant dietary fat supply elevates rates of fatty acid (FA) oxidation. The objective of this study was to determine whether an obesogenic, high-fat, sucrose-enriched (HFS) diet or a carbohydrate-free ketogenic diet (KD) exert distinct effects on fat, glucose, and ketone metabolism in oxidative and glycolytic skeletal muscles. Male Wistar rats were fed either a HFS diet or a KD for 16 weeks. Subsequently, the soleus (Sol), extensor digitorum longus (EDL), and epitrochlearis (Epit) muscles were extracted to measure palmitate oxidation, insulin-stimulated glucose metabolism, and markers of mitochondrial biogenesis, ketolytic capacity, and cataplerotic and anaplerotic machinery. Sol, EDL, and Epit muscles from KD-fed rats preserved their ability to elevate glycogen synthesis and lactate production in response to insulin, whereas all muscles from rats fed with the HFS diet displayed blunted responses to insulin. The maintenance of metabolic flexibility with the KD was accompanied by muscle-fiber-type-specific adaptive responses. This was characterized by the Sol muscle in KD-fed rats enhancing mitochondrial biogenesis and ketolytic capacity without elevating its rates of FA oxidation in comparison with that in HFS feeding. Conversely, in the Epit muscle, rates of FA oxidation were increased, whereas the ketolytic capacity was markedly reduced by the KD in comparison with that by HFS feeding. In the EDL muscle, the KD also increased rates of FA oxidation, although it did so without altering its ketolytic capacity when compared to HFS feeding. In conclusion, even though obesogenic and ketogenic diets have elevated contents of fat and alter whole-body substrate partitioning, these two dietary interventions are associated with opposite outcomes with respect to skeletal muscle metabolic flexibility.

## 1. Introduction

The suppression of glucose metabolism under conditions of an abundant dietary fat supply can be partially attributed to changes in cellular substrate partitioning. The metabolic shift that coincides with the elevation of whole-body FA oxidation and glucose intolerance has been of concern in diabetic and insulin-resistant patients [1]. Conversely, from the perspective of endurance performance, a higher rate of skeletal muscle FA oxidation could be advantageous due to its potential glycogen-sparing effect [2]. Thus, the utilization of high-fat low-carbohydrate diets in endurance athletes has recently gained attention, particularly the ketogenic diet (KD) [3] that provides an abundance of fat and ketones as alternative sources of fuel for skeletal muscles [2,4]. In fact, the skeletal muscle gene expression of *Cpt1b* [5], fat oxidation [4], and markers of the mitochondrial biogenesis [6] and fractional areas [7] were elevated, whereas glucose oxidation [4], GLUT4 and IRS1 contents, and glucose tolerance were reduced by the KD in exercise-trained individuals [8]. The KD has also been shown to significantly enhance ketone-mediated ATP production relative to glucose- or fat-mediated ATP production [4]. Thus, the abundance of fat and relatively lower content of dietary carbohydrates shift the metabolic substrate preference away from glucose and toward FAs. 

Skeletal muscles display distinct oxidative and glycogenolytic capacities [9] and activities of enzymes involved in ketone oxidation [10]. Thus, the ability of oxidative and glycolytic muscles to adjust substrate partitioning according to fat and glucose dietary abundance may also significantly differ. Indeed, Fukazawa et al. showed that a KD increased the protein content of the ketolytic enzyme 3-oxoacid CoA transferase (OXCT) in the epitrochlearis (Epit) muscle [11], whereas Shimizu et al. reported a significant attenuation of OXCT gene expression in the gastrocnemius muscle [5]. Moreover, there is evidence that the KD increases mitochondrial mass in the red gastrocnemius muscle [7], which was contrasted by reports that the KD did not alter PGC-1α levels in the extensor digitorum longus (EDL) [12] or soleus (Sol) muscles [6]. Taken together, these data point to an apparent gap in the literature where KD-induced metabolic adaptations in substrate preference in muscles of varying oxidative and glycolytic capacities are yet to be fully elucidated. In this context, this study aimed to investigate whether the obesogenic, high-fat sucrose-enriched (HFS) diet and the KD would differ in their effects on metabolic substrate selection in muscles with varying fiber-type compositions. To achieve this, we compared the metabolic responses of Sol, EDL, and Epit rat muscles to either a KD (80% fat, lard; 0% carbohydrate; 20% protein) or typical obesogenic HFS diet (60% fat, lard; 20% sucrose; 20% protein). Furthermore, previous studies comparing the KD to a control diet had significantly different protein contents [7,11,13]. Importantly, skeletal muscle atrophy has been found in studies that reduce the protein content of a KD [14], whereas studies that use a normal protein diet report the maintenance of lean mass [13]. Varying protein contents also elicit distinct metabolic adaptations. For instance, Nakao et al. used a KD that was 4.8% kcal protein, compared to the control diet that was ~14% kcal protein, and observed a decrease in gastrocnemius pyruvate dehydrogenase (PDH) activity [14]. Huang et al., on the other hand, used a KD with a similar protein level to the control diet (16%) and reported no alteration in PDH activity [13]. Therefore, it is possible that the muscle metabolic adaptations do not derive from the low-carb high-fat content of the KD itself, but rather from its low protein content. To eliminate this as a confounding variable, we equalized the protein contents in both diets. Additionally, despite the high fat content in both the obesogenic HFS diet and the KD, the latter diet has been shown to improve metabolic health [4], whereas the former is detrimental to metabolic health. This suggests that the high fat content may not by itself be the main disruptor of normal metabolic function in skeletal muscles. Finally, because the KD was devoid of carbohydrates and hepatic ketone production is enhanced by the KD [3], besides rates of FA oxidation, we also assessed markers of mitochondrial biogenesis and the content of specific enzymes involved in the ketolytic, anaplerotic, and cataplerotic machineries in skeletal muscles with distinct fiber-type compositions. Here, we provide evidence that, contrary to the HFS diet, the carbohydrate-free KD enhances rates of FA oxidation and ketolytic capacity without impairing insulin-stimulated glucose metabolism in oxidative and glycolytic skeletal muscles.

## 2. Materials and Methods

Reagents—Fatty-acid (FA)-free bovine serum albumin (BSA), glycogen, and palmitic acid were obtained from Sigma (St. Louis, MO, USA). Human insulin (Humulin R) was purchased from Eli Lilly Inc. (Toronto, ON, Canada). D-[U-^14^C]glucose and [1-^14^C]palmitic acid were acquired from GE Healthcare Radiochemicals (Quebec City, QC, Canada). The Lactate Colorimetric/Fluorometric Assay Kit was purchased from BioVision (Milpitas, CA, USA). Protease (cOmplete Ultra Tablets) and phosphatase (PhosSTOP) inhibitors were obtained from Roche Diagnostics GmbH (Mannheim, Germany). The peroxisome proliferator-activated receptor γ coactivator-1α (PGC-1α, cat. no. AB3242) antibody was purchased from Millipore (Temecula, CA, USA). The β-hydroxybutyrate (BHB; cat. No. ab83390) assay kit and the mitochondrial transcription factor A (TFAM; Cat. # ab131607), 3-oxoacid CoA-transferase 1 (OXCT; Cat. # ab224250), and acetoacetyl-CoA acetyltransferase 1 (ACAT1; Cat # ab168342) antibodies were purchased from Abcam (Toronto, ON, Canada). Pyruvate carboxylase (PC; Cat # 49381), phosphoenolpyruvate carboxykinase 1 (PCK1; Cat. # 12940), and phosphoenolpyruvate carboxykinase 2 (PCK2; 6924) antibodies were purchased from Cell Signaling (Danvers, MA, USA).

Animals and diet—Male albino rats (Wistar strain) at approximately 250 g were individually housed at 22 °C on a 12/12-h light–dark cycle. These rats were assigned either a high-fat sucrose-enriched (HFS) diet (20% kcal protein; 60% kcal fat (lard/soybean oil); 20% kcal carbohydrate (sucrose)) or KD (20% kcal protein; 80% kcal fat (lard/soybean oil); 0% kcal carbohydrate) and fed for 16 weeks ad libitum. Both diets were purchased from Research Diets (New Brunswick, NJ, USA). 

Ethics approval—The protocol containing all animal procedures described in this study was specifically approved by the Committee on the Ethics of Animal Experiments of York University (York University Animal Care Committee, YUACC, permit number: 2021-03) and performed strictly in accordance with the YUACC guidelines. Date of approval: 18 July 2023. All tissue extraction procedures were performed under ketamine/xylazine anesthesia, and all efforts were made to minimize suffering [15]. All experiments in this study were carried out in compliance with the ARRIVE guidelines [16].

Measurement of blood glucose levels—Blood from all animals in the fed state was collected between 15:00 and 16:00 h with saphenous vein bleeding. Plasma glucose was measured using a Contour Next one meter blood glucose monitoring system by Ascensia Diabetes Care US (Parsippany, NJ, USA).

Muscle isolation and incubation—All animals were anesthetized with a single intraperitoneal injection of ketamine (90 mg/100 g BW) and xylazine (10 mg/100 g BW). Subsequently, Sol, EDL, and Epit muscles were quickly extracted. The percentages of type I, type Iia, and type Iib in Sol, EDL, and Epit muscles are 84/16/0, 3/57/40 [17], and 15/20/65 [18], respectively. From these muscles, strips were cut (18–22 mg) and pinned onto thin stainless steel wire clips to maintain muscle length. Thereafter, muscle strips were immediately placed in plastic scintillation vials containing 2 mL of pre-gassed (30 min with O_2_:CO_2_-95:5% (vol/vol)) Krebs–Ringer bicarbonate (KRB) buffer containing 3.5% FA-free BSA and 5.5 mM glucose. Vials were sealed and subjected to continuous gasification for the entirety of the 1 h pre-incubation period. 

Measurement of glucose oxidation, glycogen synthesis, and lactate production—Following the pre-incubation period, one set of muscles was then transferred to vials containing 2 mL of the same KRB buffer plus D-[U-^14^C]glucose (0.2 µCi/mL) and incubated under continuous gasification for one additional hour, either in the absence (basal) or presence of insulin (100 nM) for the determination of glycogen synthesis and glucose oxidation, as previously described [19]. For lactate production, media aliquots were first deproteinated using 10 kDa filters (centrifuged at 13,000 rpm for 15 min at 4 °C) and then assayed using a colorimetric assay kit, according to the manufacturer’s instructions. 

Measurement of palmitate oxidation in isolated muscles—Palmitate oxidation was measured by assessing the production of ^14^CO_2_ from [1-^14^C]palmitic acid. Muscles were pre-incubated as previously described and then transferred to vials containing 2 mL of KRB buffer plus 0.2 mM of cold palmitic acid, previously complexed with FA-free BSA and [1-^14^C] palmitic acid (0.2 µCi/mL). The muscles were incubated under continuous gasification for 1 h, and the vials had a centered isolated well containing a loosely folded piece of filter paper moistened with 0.2 mL of 2-phenylethylamine/methanol (1:1, vol/vol). Following the incubation and acidification of the media, the filter papers were carefully removed and transferred to scintillation vials for radioactivity counting [15].

Western blotting analysis of PGC1α, TFAM, OXCT, ACAT1, PC, PCK1, and PCK2 contents in Sol, EDL and Epit muscles—Muscle samples that were not subject to incubation were flash frozen in liquid nitrogen and stored at −80 °C until the Western blotting analysis. Muscle tissues from the Sol, EDL and Epit muscles were homogenized in a buffer containing 25 mM Tris-HCl, 25 mM NaCl (pH 7.4), 1 mM MgCl_2_, 2.7 mM KCl, 1% Triton X-100, and protease and phosphatase inhibitors (Roche Diagnostics GmbH, Mannheim, Germany). The homogenates were centrifuged, the supernatant was collected, and an aliquot was used for protein determination with the Bradford method. Samples were diluted 1:1 vol:vol with a 2× Laemmli sample buffer and heated to 95 °C for 5 min. Subsequently, 25 µg of protein was loaded into each well. Samples were thereafter subjected to SDS-PAGE, transferred to a PVDF membrane, and probed for the proteins of interest. All primary antibodies were used at a dilution of 1:1000. Ponceau staining was used as a loading control. All densitometry analyses were performed using the ImageJ program, version 1.46r.

Statistical Analyses—Data were expressed as mean ± SD. Statistical analyses were performed using two-way ANOVAs with Bonferroni post hoc and Student’s *t*-tests, as indicated in the figure legends. If data were not normally distributed, or if data had an inequality of variance, Mann–Whitney and Welch’s tests were used, respectively. GraphPad Prism software version 9.1.12 was used for all statistical analyses. The level of significance was set to *p* < 0.05.

## 3. Results

Effects of diet on glycaemia and plasma βHB levels—Blood glucose levels did not differ between the HFS and KD groups at weeks 8 and 16 (Table 1). However, the KD significantly elevated plasma βHB levels 1.6- and 1.9-fold at weeks 8 and 16, respectively, relative to the HFS diet (Table 1). The concentration of βHB in the circulation changed in a time-dependent manner in KD-fed animals, being highest at week 8 (Table 1). Despite a 24% reduction in plasma βHB from weeks 8 to 16 in the KD group, week 16 levels were elevated 2.2-fold, relative to week 0 (Table 1). 

**Figure 1:**
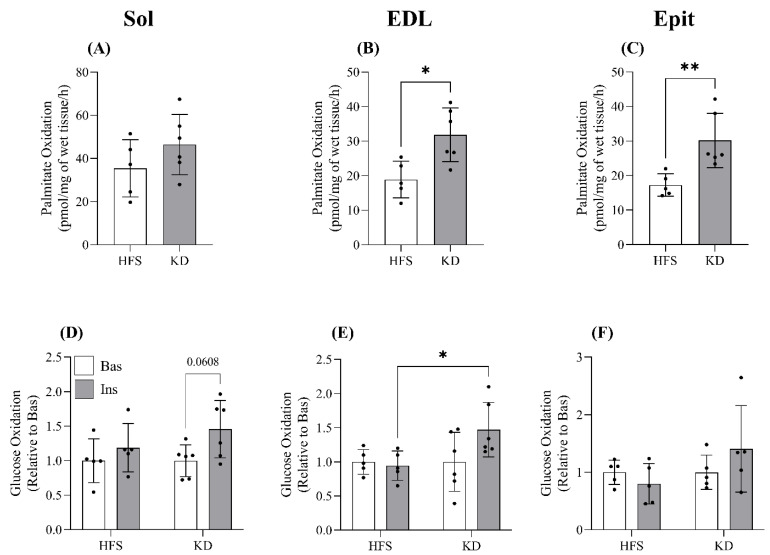
Palmitate oxidation was not significantly altered by diet in the soleus muscle (Sol, (**A**)); however, it was significantly elevated by the ketogenic diet (KD) in the extensor digitorum longus (EDL, (**B**)) and epitrochlearis (Epit, (**C**)) muscles. Conversely, glucose oxidation was not significantly enhanced in either of the two groups in any of the muscles with insulin (Ins) stimulation (**D**–**F**). Despite this, the fold increase in insulin-stimulated glucose oxidation in the EDL muscle, relative to basal (Bas) values, was enhanced by the KD (**E**). Data are expressed as mean ± SD. n = 5–6 rats. Student’s *t*-test was used to compare groups for (**A**–**C**), whereas a two-way ANOVA with Bonferroni post hoc comparisons was used for (**D**–**F**). * *p* < 0.05, ** *p* < 0.01.

Effects of diet on palmitate and glucose oxidation in skeletal muscle—Sol palmitate oxidation did not significantly differ between the HFS- and KD-fed rats (Figure 1A); however, in both the EDL and Epit muscles, the KD increased palmitate oxidation ~1.7-fold in comparison with the HFS diet (Figure 1B and C, respectively). These muscle-specific adaptive responses were also observed with respect to glucose oxidation. In the Sol, EDL, and Epit muscles in HFS-fed animals, insulin-stimulated glucose oxidation was blunted (Figure 1D–F). In the KD-fed rats, the rates of insulin-stimulated glucose oxidation in the Sol, EDL, and Epit muscles were higher than the basal values, but did not reach statistical significance (Figure 1D–F). Only the EDL muscles in the KD-fed rats displayed rates of insulin-stimulated glucose oxidation that were significantly higher than those in the HFS-fed rats (Figure 1E). Thus, whereas the KD enhanced FA oxidation in a muscle-specific manner, insulin-stimulated glucose oxidation was similarly impaired in all muscles.

**Figure 2:**
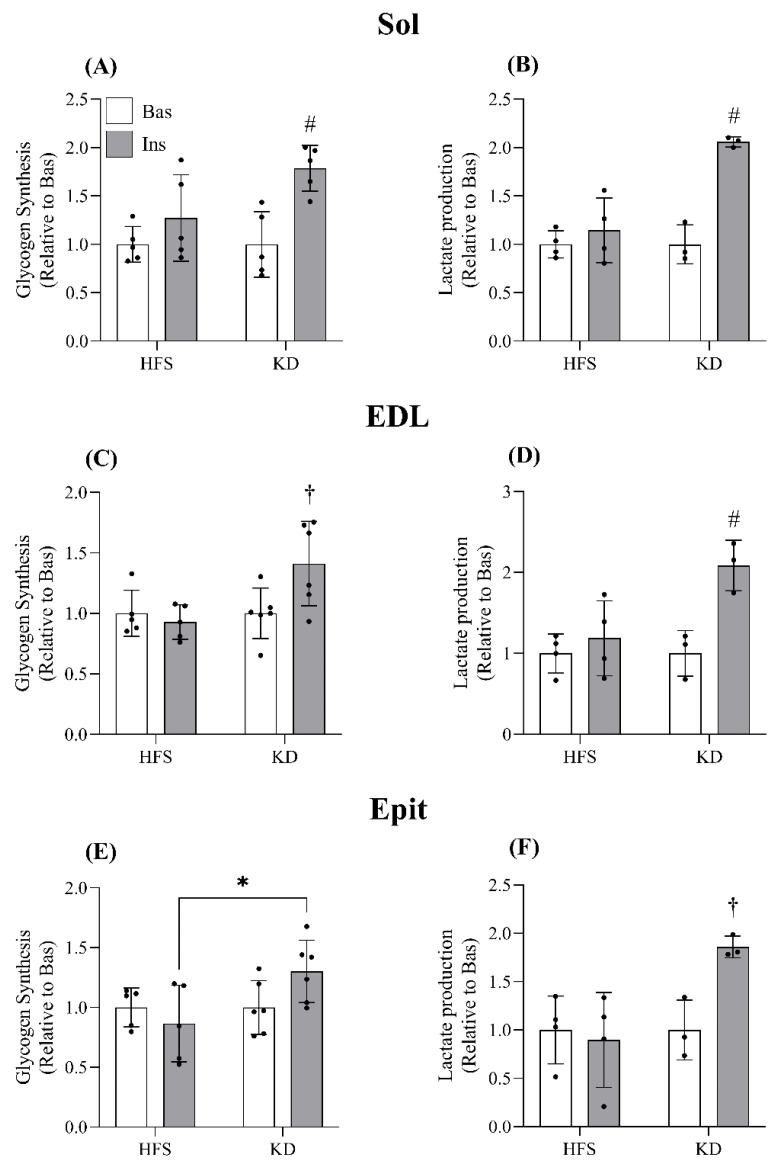
Ins stimulation increased glycogen synthesis in the Sol and EDL muscles of KD-fed rats (**A** and **C**, respectively). Epit glycogen synthesis did not increase relative to Bas values in either of the two groups; however, the fold increase in Ins-stimulated glycogen synthesis was greater with KD (**E**). Similarly, Ins significantly enhanced lactate production in all three muscles of KD-fed rats (**B**,**D**,**F**). Data are expressed as mean ± SD. n = 3–6. A two-way ANOVA with Bonferroni post hoc tests was used to compare the groups. * *p* < 0.05; ^#^ *p* < 0.05 vs. all other groups; ^†^ *p* < 0.05 vs. HFS Ins and KD Bas.

Effects of diet on insulin-stimulated glycogen synthesis and lactate production—Insulin-stimulated rates of glycogen synthesis were blunted in the Sol, EDL, and Epit muscles in rats fed with the HFS diet (Figure 2A, C and E, respectively), whereas in the Sol and EDL muscles of KD-fed rats, insulin increased this variable 1.8-fold (Figure 2A) and 1.5-fold (Figure 2C), respectively, in comparison with basal values. In Epit muscles from KD-fed rats, insulin stimulation did not significantly elevate glycogen synthesis in comparison to basal values; however, the glycogen synthesis response to insulin was significantly higher than that of the HFS-fed rats (Figure 2E). Insulin-stimulated lactate production, an indication of glycolytic capacity [20], was also blunted in the Sol, EDL, and Epit muscles from HFS-fed rats; whereas in the KD-fed rats, the Sol, EDL, and Epit muscles incubated with insulin displayed 2.1-, 2.1-, and 1.9-fold increases in lactate production in comparison to basal values (Figure 2B, D and F, respectively). Therefore, the KD preserved the capacity of oxidative and glycolytic muscles to store glycogen and perform glycolysis when stimulated with insulin.

**Figure 3:**
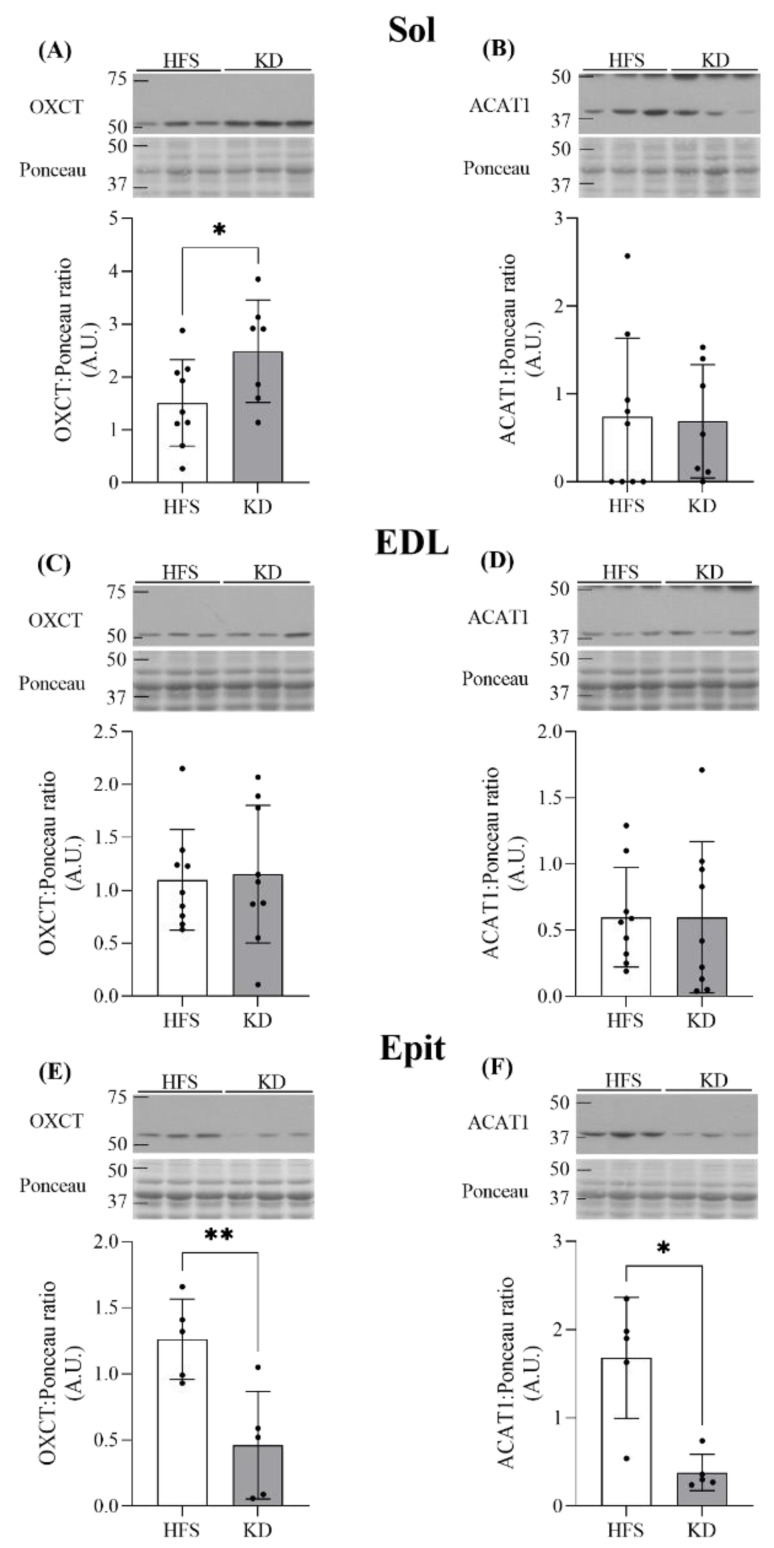
Sol OXCT content was significantly elevated by the KD (**A**), whereas Sol ACAT1 was unaffected by diet (**B**). Neither diet altered EDL muscle contents of OXCT or ACAT1 (**C** and **D**, respectively). Conversely, OXCT and ACAT1 contents were attenuated in the Epit muscle of KD-fed animals (**E** and **F**, respectively). Data are expressed as mean ± SD. n = 5–9. Student’s *t*-test was used to compare groups, except for (**B**) and (**F**), where the Mann–Whitney test was used. * *p* < 0.05, ** *p* < 0.01.

Effects of the KD on the contents of ketolytic enzymes OXCT and ACAT1 in Sol, EDL, and Epit muscles—Because the circulating βHB levels were elevated in KD-fed rats compared with those of the HFS-fed rats, it was possible that the effects of ketones on substrate partitioning in skeletal muscles would also be different between the dietary interventions and fiber-type compositions. To assess that possibility, we measured the ketolytic enzymes OXCT and ACAT1 in Sol, EDL, and Epit muscles from rats fed either with the HFS diet or KD. We found that the OXCT content increased 1.6-fold in the Sol muscles of KD-fed animals (Figure 3A), whereas ACAT1 levels were unaltered by either of the two diets in this highly oxidative muscle (Figure 3B). In the EDL, OXCT and ACAT1 contents did not differ between the HFS- and KD-fed rats (Figure 3C and D, respectively). In contrast, the Epit OXCT and ACAT1 contents were 63% and 77% lower, respectively, in KD-fed animals when compared to those in the HFS group (Figure 3E and F, respectively). Therefore, in comparison to the HFS diet, the KD enhanced and suppressed the ketolytic capacity of the Sol and Epit muscles, respectively, whereas in the EDL muscle, the ketolytic capacity was unaffected by either the HFS diet or KD.

**Figure 4:**
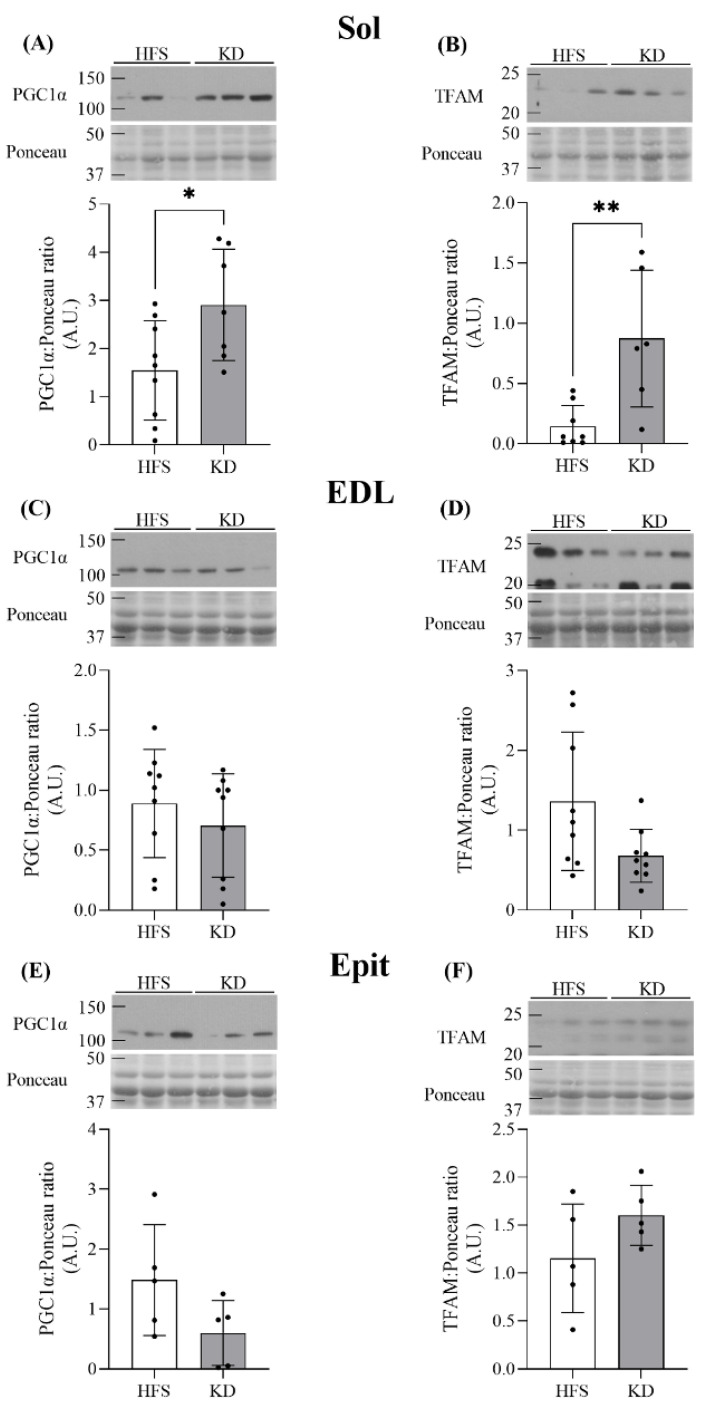
The Sol muscle demonstrated KD-induced elevations in PGC1α and TFAM (**A** and **B**, respectively), whereas diet did not alter the contents of either of these proteins in either the EDL (**C** and **D**) or Epit (**E** and **F**) muscles. Data are expressed as mean ± SD. n = 5–9. Student’s *t*-test was used to compare groups, except for (**B**) and (**D**), where the Mann–Whitney test and Welch’s *t*-test were used, respectively. * *p* < 0.05, ** *p* < 0.01.

Effects of diet on PGC-1α and TFAM protein levels in Sol, EDL, and Epit muscles—The muscle-specific differences in substrate preference could be attributed to distinct dietary effects on mitochondrial biogenesis. Thus, we measured the contents of PGC-1α and TFAM, which are markers of mitochondrial biogenesis and content [21]. In Sol muscles, despite not increasing palmitate oxidation, the KD significantly elevated PGC-1α and TFAM contents 1.9- and 6-fold, respectively (Figure 4A and B, respectively). However, in the EDL (Figure 4C and D) and Epit (Figure 4E and F) muscles, PGC-1α or TFAM levels did not differ between the HFS diet and KD. 

**Figure 5:**
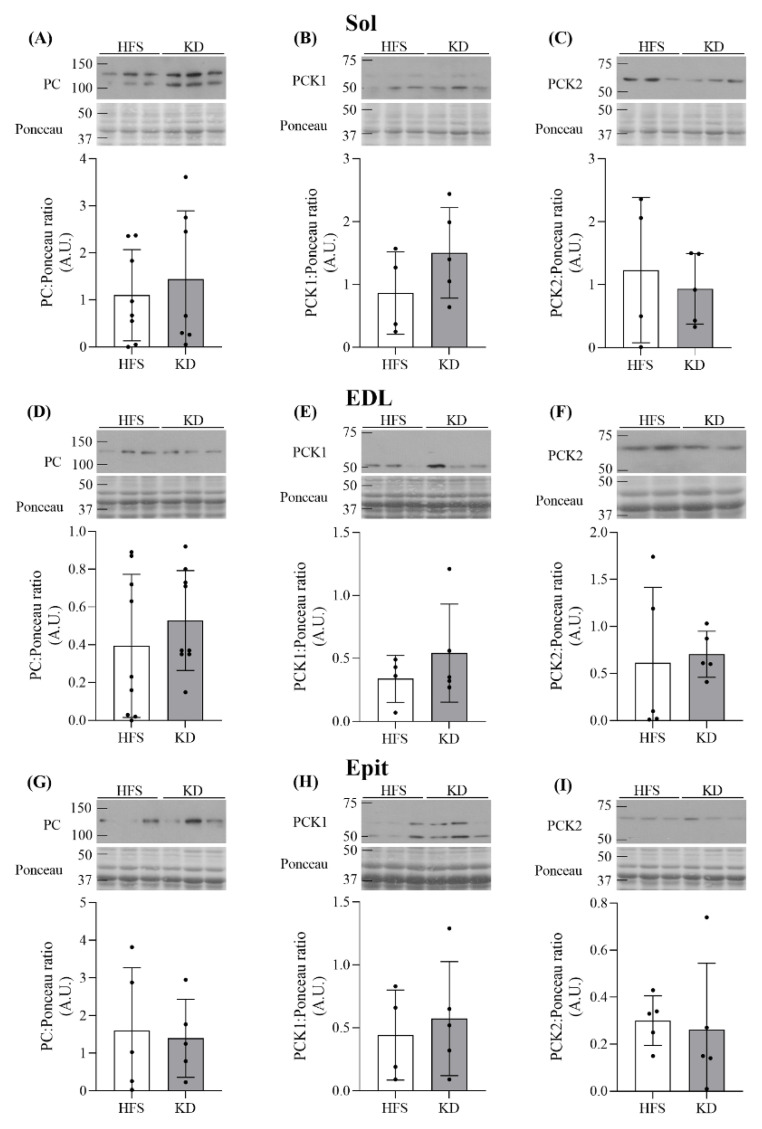
PC, PCK1, and PCK2 were not altered by diet in the Sol (**A**–**C**, respectively), EDL (**D**–**F**, respectively) or Epit (**G**–**I**, respectively) muscles. Data are expressed as mean ± SD. n = 4–9. Student’s *t*-test was used to compare groups, except for (**E**), where the Mann–Whitney test was used.

Effects of the KD on PC, PCK1, and PCK2 levels in Sol, EDL, and Epit muscles—Substrate oxidation is dependent on the availability of tricarboxylic acid (TCA) cycle intermediates, which is largely controlled by cataplerotic and anaplerotic enzymes [22]. To investigate whether the shift toward FA oxidation induced by both diets, and particularly the carbohydrate-free KD, could be attributed to this, we assessed the contents of the anaplerotic protein PC, and the cataplerotic PCK1 and PCK2 [22,23]. Despite differences in oxidative and glycolytic capacities among the three muscles, no differences were found in the protein levels of PC, PCK1, and PCK2 in the Sol (Figure 5A–C), EDL (Figure 5D–F), or Epit (Figure 5G–I) muscles when comparing the HFS diet and KD. Thus, the contents of anaplerotic and cataplerotic enzymes in oxidative and glycolytic muscles did not contribute to the muscle-specific diet effects on substrate oxidation.

## 4. Discussion

The main finding of this study was that despite inducing a higher rate of FA oxidation than the HFS diet, the KD preserved the ability of oxidative and glycolytic muscles to elevate glucose metabolism in response to insulin. Thus, our data provided compelling evidence that it is the chronic exposure to a combination of high fat and sugar, rather than solely to the elevated fat content of the diet, that disrupts the ability of skeletal muscles to process glucose in response to insulin. This was supported by elevated rates of insulin-stimulated glycogen synthesis and lactate production in the Sol, EDL, and Epit muscles in the KD-fed rats, which contrasted with the blunted responses displayed by these muscles in rats fed with the HFS diet. The analysis of insulin-stimulated glucose oxidation also revealed a similar trend when comparing both diets, although with KD eliciting a much less pronounced and fiber-type-specific effect on this variable than on rates of insulin-stimulated glycogen synthesis and lactate production. Because Sol muscles are rich in Type I fibers [17] and normally display higher capacities for FA oxidation when exposed to an HFS diet [13,15,24], the ability of these muscles to further elevate glucose oxidation when exposed to the KD may have been limited. Also, KD-fed Sol muscles were the only ones eliciting higher levels of the ketolytic enzyme OXCT than the HFS-fed counterparts, which likely enhanced the capacity of the Sol muscles to oxidize ketones. Thus, in Sol muscles, more glucose was diverted towards other pathways of glucose metabolism when stimulated with insulin as compared to the EDL and Epit muscles. Indeed, the rates of insulin-stimulated glycogen synthesis and lactate production in the Sol muscle were the highest among the muscles studied. 

In well-trained cyclists habituated to a KD, GLUT4 and IRS1 protein contents were decreased in vastus lateralis muscles, which is consistent with the whole-body glucose intolerance found in these athletes [8]. KD-induced glucose intolerance has previously been attributed to attenuated β-cell mass in the pancreas [25]. In the present study, we found that both glycogen synthesis and lactate production were preserved in the EDL of KD-fed rats, as indicated by the sensitivity of this muscle to insulin. Moreover, the elevation of glucose oxidation in the EDL muscle in response to insulin was significantly higher in the KD-fed rats than that in the HFS-fed rats. Together, these findings indicated that the KD leads to a disuse-mediated adaptation in pancreatic cell function that renders the organism less tolerant to a bolus of glucose. However, in line with the preserved response to insulin in the EDL, this mechanism is not indicative of disease and does not impact the capacity of skeletal muscles to metabolize glucose. In fact, despite the muscle-specific shift in substrate partitioning toward fatty acid oxidation and enhanced ketolytic capacity, all three skeletal muscles in the KD group demonstrated an enhanced ability to store glycogen and use glucose when exposed to insulin. Conversely, muscles from the HFS group displayed the classical impairment in insulin-stimulated glucose handling.

As expected, the KD significantly enhanced blood ketone levels, albeit not to the same extent as reported in other rodent KD studies [12,14,25]. This could be explained by the difference in the macronutrient composition of the KD used. In the present study, the protein content of the diet was 20% calories, whereas in studies showing a higher induction of ketosis, the protein contents ranged from 4.8–10% calories [12,14,25]. This is an important distinction, given that decreasing the protein content of a KD has been shown to significantly increase βHB levels in rats [26]. Furthermore, the apparent attenuation in βHB from weeks 8 to 16 points to a KD-induced adaptation, whereby ketolytic tissues, including those in the Sol muscle, enhanced their metabolic capacities for ketones, which likely led to an attenuation in the circulating levels of this substrate [2].

It was surprising to find that despite increasing PGC-1α and TFAM, the KD did not increase palmitate oxidation in Sol muscles, when compared to the HFS diet. Conversely, in EDL and Epit muscles, PGC-1α and TFAM protein levels did not differ between the KD- and HFS-fed rats, yet the rates of FA oxidation in the former diet were significantly higher than those in the latter. In this context, despite increasing the proteins involved in mitochondrial biogenesis, the 20% additional fat content of the KD in comparison to that of the HFS diet did not further elevate the FA oxidative capacity of Sol muscles. Instead, the induction of mitochondrial biogenesis in the Sol muscle could also have served to increase the utilization of circulating ketone bodies as a fuel source, which is consistent with the KD-induced elevation in Sol muscles of OXCT protein levels, a major rate-limiting ketolytic enzyme [27]. In line with this, the administration of a ketone ester drink prior to exercise to endurance-trained athletes suppressed glycolysis and increased reliance on ketones for energy in the vastus lateralis muscle [28]. Thus, it is plausible that in the current study, ketones displaced glucose as an oxidative substrate in the Sol muscles of KD-fed rats, which could further attenuate the reliance on glucose and explain the modest insulin-stimulated glucose oxidation response in all three muscles from KD-fed rats. Conversely, the KD-induced enhancement in glycolytic capacity likely served to replenish oxaloacetate to support fat and ketone oxidation in the absence of dietary carbohydrate intake [22]. However, oxaloacetate availability for complete oxidation of acetyl-CoA produced from ketone bodies may also have been provided by acetoacetate activation via the conversion of succinyl-CoA to succinate in the TCA cycle, with the latter being converted to oxaloacetate through the subsequent reactions of the TCA cycle [29]. This likely facilitated the entry of more acetyl-CoA into the cycle without causing alterations to the levels of anaplerotic or cataplerotic enzymes. 

Even though the Epit is a highly glycolytic muscle [18], its rates of FA oxidation were significantly higher in the KD rats than in the HFS-fed animals. This was surprising given that both diets were high in fat. In fact, the HFS diet had already been show to enhance Epit FA oxidation in comparison to standard chow [15,24]. Thus, this study provided evidence that the absence of carbohydrates in the KD potentiated the reliance of the Epit muscle on fat as an oxidative substrate. Interestingly, this occurred independently of alterations in mitochondrial biogenesis proteins and, also, by marked reductions in the levels of the ketolytic enzymes OXCT and ACAT1, suggesting that Epit muscles enhanced their capacities to oxidize fat at the expense of ketones. 

The EDL muscle displayed an intermediate effect relative to the Sol and Epit muscles. Like in the Epit muscle, palmitate oxidation was enhanced, whereas markers of mitochondrial biogenesis were unaltered. In line with this, KD feeding enhanced the aerobic capacity of the EDL muscle, despite not altering PGC1α content [12]. Moreover, other muscles with mixed-fiber-type compositions [17], including the gastrocnemius [5,13] and vastus lateralis [4] muscles, showed a KD-induced upregulation of fat oxidative capacity. However, unlike the Epit muscle, ketolytic capacity in the EDL muscle did not differ between the two dietary interventions. The preserved ketolytic capacity in the EDL versus Epit muscles, despite the similar enhancement in fat oxidation, could be attributed to the relatively higher oxidative capacity of the former [17], and therefore, the ability to perform FA oxidation without altering ketolytic capacity. Nevertheless, in the present study, we observed that only fat oxidation was increased in the EDL muscle, suggesting that the upregulation of mitochondrial biogenesis may be required to enhance the ketone-oxidizing capacity of the EDL muscle when under conditions of abundant dietary FA availability.

## 5. Conclusions

In conclusion, despite eliciting a higher rate of FA oxidation than the obesogenic HFS diet, the carbohydrate-free KD preserved the ability of oxidative and glycolytic muscles to elevate glucose metabolism in response to insulin. Thus, it is the chronic exposure to a combination of high fat and sugar, rather than solely to the elevated fat content of the diet, that disrupts the ability of skeletal muscles to process glucose in response to insulin. This was characterized by the KD-induced elevation in mitochondrial biogenesis and ketolytic capacity in the Sol muscle, despite the lack of alteration in FA oxidation relative to that in the HFS group. In contrast, Epit FA oxidation was significantly elevated with KD feeding, whereas proteins involved in ketolysis were markedly reduced. Similarly, FA oxidation was increased in the EDL muscle of KD-fed rats, although the ketolytic capacity was unaltered in this muscle with the KD, relative to that in the HFS-fed rats. Lastly, the levels of anaplerotic and cataplerotic enzymes in all muscles studied did not differ, even though the KD was devoid of carbohydrates. Collectively, these findings indicated that, contrary to the obesogenic HFS diet, the carbohydrate-free KD maintained skeletal muscle metabolic flexibility while also enhancing, in a muscle-specific manner, rates of FA oxidation and ketolytic capacity.

## Figures and Tables

**Table 1:** Blood glucose and plasma beta-hydroxybutyrate (βHB) levels at weeks 0, 8, and 16 of dietary intervention. Blood glucose levels were not different between the two dietary groups; however, βHB was elevated in the KD group at weeks 8 and 16, relative to that in the HFS group.

		Week		
		0	8	16
Glycaemia	HFS	7.31 ± 0.54	7.19 ± 0.47	6.77 ± 0.72 ^‡^
(mM)	KD	7.29 ± 0.35	6.99 ± 0.34	6.57 ± 0.43 ^‡^
βHB	HFS	0.16 ± 0.06	0.24 ± 0.08	0.15 ± 0.04
(mM)	KD	0.13 ± 0.04	0.38 ± 0.08 ^#^	0.29 ± 0.05 ^†^

^#^ *p* < 0.05 vs. week 0 KD and week 8 HFS; ^†^ *p* < 0.05 vs. week 0 KD, week 8 KD, and week 16 HFS. ^‡^ *p* < 0.05 vs. week 0. Data are expressed as mean ± SD. Mixed-effect model analysis. N = 7–15 for glycaemia. N = 6–7 for βHB.

## Data Availability

All data supporting the results are included within the figures revealing their range and distribution.
